# A Case of Newly Diagnosed Klippel Trenaunay Weber Syndrome Presenting with Nephrotic Syndrome

**DOI:** 10.1155/2015/704379

**Published:** 2015-04-27

**Authors:** Egemen Cebeci, Secil Demir, Meltem Gursu, Abdullah Sumnu, Mehmet Yamak, Barıs Doner, Serhat Karadag, Sami Uzun, Ahmet Behlul, Oktay Ozkan, Savas Ozturk

**Affiliations:** ^1^Department of Nephrology, Haseki Training and Research Hospital, 34087 Istanbul, Turkey; ^2^Department of Internal Medicine, Haseki Training and Research Hospital, 34087 Istanbul, Turkey

## Abstract

Klippel Trenaunay Weber syndrome (KTWS) is a rare disease characterized by hemihypertrophy, variceal enlargement of the veins, and arteriovenous (AV) malformations. Renal involvement in KTWS is not known except in rare case reports. Herein, we present a case of KTWS with nephrotic syndrome. A 52-year-old male was admitted due to dyspnea and swelling of the body for the last three months. The pathological physical findings were diffuse edema, decreased lung sounds at the right basal site, increased diameter and decreased length of the left leg compared with the right one, diffuse variceal enlargements, and a few hemangiomatous lesions on the left leg. The pathological laboratory findings were hypoalbuminemia, hyperlipidemia, increased creatinine level (1.23 mg/dL), and proteinuria (7.6 g/day). Radiographic pathological findings were cystic lesions in the liver, spleen, and kidneys, splenomegaly, AV malformation on the left posterolateral thigh, and hypertrophy of the soft tissues of the proximal left leg. He was diagnosed to have KTWS with these findings. Renal biopsy was performed to determine the cause of nephrotic syndrome. The pathologic examination was consistent with focal segmental sclerosis (FSGS). He was started on oral methylprednisolone at the dosage of 1 mg/kg and began to be followedup in the nephrology outpatient clinic.

## 1. Introduction

Klippel Trenaunay Weber syndrome (KTWS) is a rare idiopathic disease characterized by hemihypertrophy of the bones and soft tissues, variceal enlargement of the veins in the involved extremity, and arteriovenous (AV) malformations. The disease was first described by Klippel and Trenaunay as Klippel Trenaunay syndrome (KTS) which included hemihypertrophy and varices in 1900 after which Weber called the disease Klippel Trenaunay Weber syndrome with the addition of AV malformations in 1907 [1, 2]. The pathogenetic mechanism of the increased angiogenesis is thought to be mutations in the angiogenic factor (VG5Q) gene via transcription and increased activity [3]. VG5Q gene has been identified in blood vessels, is secreted during angiogenesis, and increases endothelial cell proliferation. The involvement is unilateral typically, of the lower extremity in 95%, upper extremity in 5%, and both lower and upper extremities in 15% of the cases [4]. Capillary lesions are associated with soft tissue swelling and bone hypertrophy. Patients with this syndrome have a wide spectrum of presentation from asymptomatic disease to life-threatening bleeding and embolism.

The symptoms appear before the age of ten in about 75% of cases in this congenital disease [5]. Although the treatment strategy is conservative unless complicated, patients need close orthopedic follow-up since the length of lower extremities differs frequently [6]. The differential diagnosis of KTWS includes KTS, Maffucci syndrome, Proteus syndrome, and other capillary malformations not associated with any syndrome [7].

Renal involvement in KTWS is not known except in rare case reports. Herein, we present a case of KTWS diagnosed at the age of 52 together with nephrotic syndrome.

*Key Message*. The diagnosis of Klippel Trenaunay Weber syndrome may be delayed into adulthood and FSGS may coexist with this syndrome.

## 2. Case Presentation

**Figure 1:**
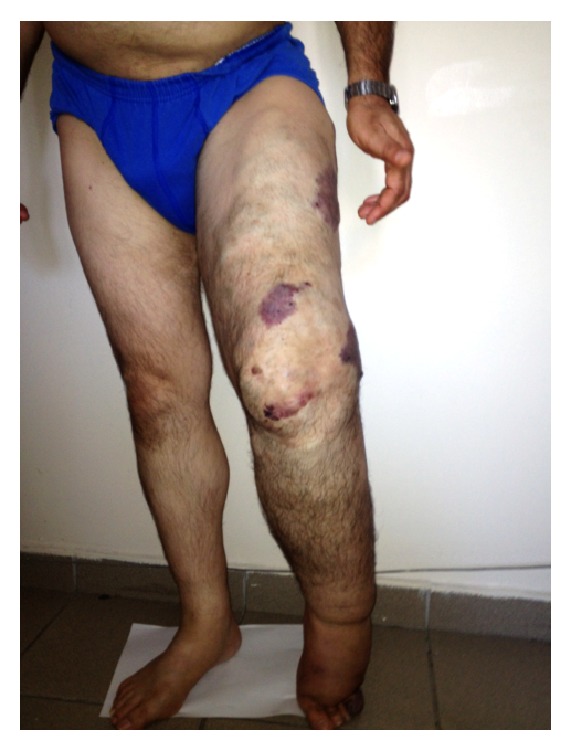
The appearance of the lower extremities of the patient showing shortness and thickness of the left leg with variceal enlargement of the veins and hemangiomas.

A 52-year-old male was admitted to the outpatient clinic of the department of internal medicine with the complaints of progressively increasing dyspnea and swelling of the body during the last three months. The patient had variceal enlargements of the veins from the time of birth and his left leg was thicker than the right one, but he did not have a certain diagnosis. The family history was negative. The pathological findings on physical examination at the time of diagnosis were diffuse edema in the body, decreased lung sounds at the right basal site, increased diameter and decreased length of the left leg compared with the right one, diffuse variceal enlargements, and a few hemangiomatosis lesions on the left leg (Figure 1). The pathological laboratory findings were as follows: serum albumin: 2.2 g/dL, total cholesterol: 216 mg/dL, LDL cholesterol: 152 mg/dL, creatinine: 1.23 mg/dL, and proteinuria: 7.6 g/day. Urine sediment was inactive. The abdominal ultrasonography revealed a cystic lesion of 7 × 4.5 cm diameters in the liver with thin septations and dense content in some areas; splenomegaly (133 mm), a solid lesion resembling hemangioma measured 3 × 2.5 cm at the lower pole of the spleen; and multiple anechoic cysts measured at most 2 cm at the upper pole of the spleen. The sizes of the kidneys were normal, while the echogenicity was increased. There were one cortical cyst (2.5 cm) in the upper pole of the right kidney and two cysts (the bigger one measured 6 cm in diameter) in the upper pole of the left kidney. Dynamic magnetic resonance imaging of the abdomen with intravenous contrast material showed a cystic lesion measured 53 × 47 mm with peripheral capsular contrast involvement in the segment 4a-7 of the liver and nodular lesions consistent with hemangiomas in segments 7-8 and 4A of the liver. There were also splenomegaly (136 mm), heterogeneity of the splenic parenchyma, and multiple lesions resembling hemangiomas measuring 25 mm at most in the spleen. There were simple cortical cysts in the kidneys, one in the upper pole of the right kidney (27 mm) and two in the upper pole of the left kidney (the bigger one measuring 6 mm). The radiologists reported bilateral pleural effusion reaching 15 mm of thickness on the right side and AV malformation on the left posterolateral thigh that fills the mesorectum and hypertrophy of the soft tissues of the proximal left lower extremity.

**Figure 2:**
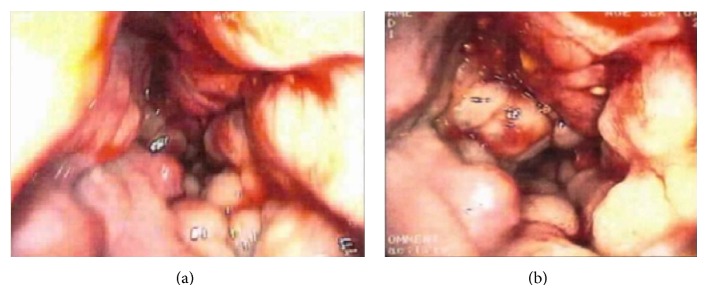
Colonoscopic findings. Diffuse blue-purple variceal enlargements on the rectal mucosa (hemangioma) and a polyp in the rectum with a diameter of 1 cm (a). The rectal mucosa was bluish purple from the 10th cm to 20th cm (hemangioma) (b).

He was diagnosed to have KTWS putting together the hemihypertrophy, diffuse variceal enlargements of the veins, and AV malformations detected radiologically. Gastroscopic examination was normal, while colonoscopy revealed diffuse blue-purple variceal enlargements on the rectal mucosa (hemangioma) and a polyp in the rectum with a diameter of 1 cm. The rectal mucosa was bluish purple from the 10th cm to 20th cm (hemangioma) (Figure 2).

**Figure 3:**
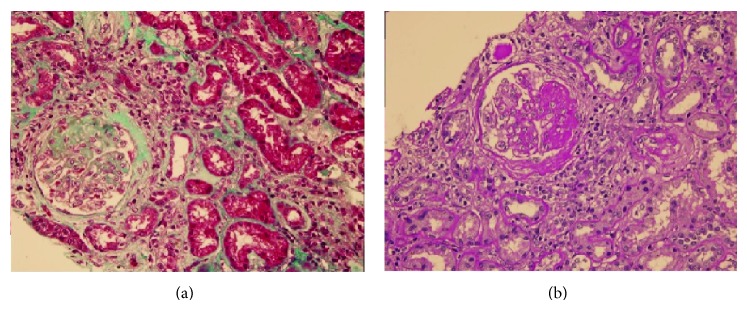
Segmental sclerotic lesion in the glomerulus. (a) Masson trichrome. (b) Periodic acid Schiff staining.

He was transferred to the nephrology clinic for evaluation of the cause of nephrotic syndrome. Paleness of the temporal regions and increased deepness of the optic hollow were detected at eye examination. Hepatitis serology, antineutrophil cytoplasmic antibody, and antinuclear antibody were negative. Complement levels were within normal limits. Renal biopsy was performed to determine the cause of nephrotic syndrome. Of the 13 glomeruli detected, five were globally sclerotic while another five had segmental sclerosis. There was prominent mesangial enlargement in other glomeruli together with patchy atrophy of tubuli, interstitial fibrosis, mild mononuclear cellular infiltration, and thickened arteriolar walls (Figure 3). No accumulation was detected with examination by immune fluorescence techniques. Electron microscopic examination was not available. With these findings, he was diagnosed as focal segmental sclerosis (FSGS). He was started on oral methylprednisolone at the dosage of 1 mg/kg and began to be followed up in the nephrology outpatient clinic. It was learned that he was admitted to the emergency clinic of another hospital due to profuse rectal bleeding at the end of the third week of steroid treatment. The steroid treatment was terminated at that time. Proteinuria was measured as 5.2 g/day and serum creatinine was 2.1 mg/dL. He is still under follow-up with conservative treatment.

## 3. Discussion

Klippel Trenaunay Weber syndrome is a rare vascular abnormality characterized by hemihypertrophy, variceal veins, and AV malformations. It is usually sporadic as in our case, although few familial cases have been reported [8]. It is a congenital disease diagnosed usually in childhood, but it should be kept in mind that there may be cases undiagnosed until adulthood as in our case.

The disease involves usually the lower extremity like our case although the trunk or face may be affected unilaterally. Hemangiomas may be limited to the skin or may be seen in bones, muscles, and solid organs. The patient we present had hemangiomas in the liver, spleen, and rectum besides the lower extremity. Variceal veins appear in the first years of life and increase in dimensions until adolescence [7]. They may cause pain, lymphedema, thrombophlebitis, and skin ulcerations. Hemihypertrophy presents either as increased length of the bones or as increased diameter due to soft tissue involvement. Hemihypertrophy is present at birth and progresses until adolescence at which time it ceases to progress. The presented case had hypertrophy of both bone and soft tissue. There may be eye abnormalities in KTWS including vascular pathologies of optic nerve, iris, choroid, retina, and orbit. The pathological findings detected at the examination of the presented case were thought to be related with KTWS.

Renal involvement in KTWS was presented as case reports of aneurysm of the renal artery, renal hemangiomas, and hydronephrosis [9–12]. A case with KTWS and renal failure was reported of which the renal biopsy showed abnormal accumulation in the mesangial tissue [13]. One case with KTWB and proteinuria was reported [14], but no data has been found in the literature about the coexistence of KTWS and FSGS.

FSGS, although usually primary, may develop as secondary due to various reasons. Among the secondary reasons, hemodynamic factors, hyperfiltration, and renal vasodilation are the major ones. The presence of acute or subacute nephrotic syndrome together with hypoalbuminemia is consistent with primary FSGS. Secondary FSGS is usually characterized by low grade proteinuria and normal serum albumin levels [15, 16]. Regarding these clinical differences between primary and secondary FSGS, the presented case was thought to be primary due to acute onset, nephrotic range proteinuria, and hypoalbuminemia. So he was started on steroid treatment. Otherwise, it was not possible to make a pathophysiological link between KTWS and FSGS present in this case.
